# FaaPred: A SVM-Based Prediction Method for Fungal Adhesins and Adhesin-Like Proteins

**DOI:** 10.1371/journal.pone.0009695

**Published:** 2010-03-15

**Authors:** Jayashree Ramana, Dinesh Gupta

**Affiliations:** Structural and Computational Biology Group, International Centre for Genetic Engineering and Biotechnology (ICGEB), Aruna Asaf Ali Marg, New Delhi, India; University of California Riverside, United States of America

## Abstract

Adhesion constitutes one of the initial stages of infection in microbial diseases and is mediated by adhesins. Hence, identification and comprehensive knowledge of adhesins and adhesin-like proteins is essential to understand adhesin mediated pathogenesis and how to exploit its therapeutic potential. However, the knowledge about fungal adhesins is rudimentary compared to that of bacterial adhesins. In addition to host cell attachment and mating, the fungal adhesins play a significant role in homotypic and xenotypic aggregation, foraging and biofilm formation. Experimental identification of fungal adhesins is labor- as well as time-intensive. In this work, we present a Support Vector Machine (SVM) based method for the prediction of fungal adhesins and adhesin-like proteins. The SVM models were trained with different compositional features, namely, amino acid, dipeptide, multiplet fractions, charge and hydrophobic compositions, as well as PSI-BLAST derived PSSM matrices. The best classifiers are based on compositional properties as well as PSSM and yield an overall accuracy of 86%. The prediction method based on best classifiers is freely accessible as a world wide web based server at http://bioinfo.icgeb.res.in/faap. This work will aid rapid and rational identification of fungal adhesins, expedite the pace of experimental characterization of novel fungal adhesins and enhance our knowledge about role of adhesins in fungal infections.

## Introduction

Adhesins are cell surface proteins that confer upon the microbes the ability of attachment to cells, tissues and/or abiotic surfaces. Adhesins pose as the first line of pathogen's stratagem of host cell invasion and are therefore indispensable determinant of its virulence. Due to importance in host cell invasion, adhesins are the subject of intense investigation to exploit its therapeutic potential. Apart from host cell attachment and mating, fungal adhesins are implicated in numerous other functions like social aggregation, foraging, biofilm formation on tissues, biomedical prosthesis and catheters [1] and xenotypic interactions with other microbes [2], [3]. Biofilm formation further contributes to increased drug resistance and persistence of infections. Differences in adhesion have been shown to be responsible for greater virulence/pathogenicity of one strain compared to the other in fungi [4], [5]. The phenotypic variability and plasticity of adhesins poses as a remarkable stress-defense mechanism for fungi allowing them to alter their adhesion properties in response to different environments [6].

Most fungal adhesins have a modular structure consisting of an N-terminal carbohydrate or peptide-binding domain, central Ser- and Thr- rich glycosylated domains and C-terminal region that mediates covalent cross-linking to the wall through modified glycosylphosphatidylinositol (GPI) anchors [6], [7]. However there are many examples that do not conform to this general model, such as Mam3, Map4 (both from *Schizosaccharomyces pombe*), WI-1/Bad1 (from *Blastomyces dermatiditis*), Int1p (*Candida albicans*) etc., rendering their identification a challenging task.

Separation and purification of such highly glycosylated proteins like adhesins by experimental techniques is an arduous task. For fungi with a diploid genome like *Candida albicans*, forward genetic approaches involving the generation of non-adhesive mutants, are also precluded [8]. Consequently, as compared to bacteria, very few adhesins have been identified in fungi.

The efficacy of anti-adhesion therapy in treating microbial infections and crop protection has been unequivocally demonstrated in several different studies [9], [10]. Microbial adhesins are immunizing components in several approved vaccine formulations and are also being currently evaluated in different organisms. There are plenty of such examples for bacteria like FHA, pertactin in B. pertussis [11], FimH for pathogenic *E*. *coli* [12], PsaA for pneumococcal disease [13], BabA for *H. pylori* [14], for protozoa like MIC1, MIC3, MIC4 in *T. gondii*, RAP-1 in *B. bovis*, CSL in *C. parvum*, BAEBL, MAEBL in *P. falciparum* [15] and in fungi like WI-1 for *B. dermatiditis* [16], Als1p, Als3p [17] and phospho-mannan adhesin (US patent 5578309 by Cutler and Han, *Candida albicans* phosphomannoprotein adhesion as a vaccine, The Research and Development Institute, Inc., 1996) against *Candida*.

The cost, time and the incumbent limitations of experimental methods, coupled with the tremendous biological significance and mounting interest in these proteins have motivated attempts to develop computational algorithms to identify adhesins. Two such algorithms are Software Program for prediction of Adhesins and Adhesin-like proteins using Neural networks (SPAAN) [18] and Malarial Adhesins and Adhesin-like proteins Predictor (MAAP) [19]. The latter is exclusively for the identification of malarial adhesins. SPAAN has been used for the genome-scale identification of fungal adhesins in one study [20], though it is trained primarily on bacterial adhesins. This prompted us to check its potential for prediction of fungal adhesins in general, and we observed that the program could not identify 38% of the 75 fungal adhesins (used as positive training set in this work, see Materials and Methods) with high confidence (i.e. these achieved P_ad_ scores below threshold of 0.7). This was reasonable since the program was trained primarily on bacterial adhesins though in the non-adhesin set, almost one-third of the proteins were from *S. cerevisiae*.

Several fungal species e.g. *Candida* spp., *Aspergillus* spp. pose serious health hazards, causing persistent infections against which there are only limited therapeutic options. Identification of adhesion molecules would further our understanding of host-tissue adhesion in fungi, thereby aiding the exploration of novel anti-fungal drug targets and vaccine candidates. In this direction, we present a SVM based method aimed at facilitating the identification of fungal adhesins.

## Results

### Performance of similarity-based searches

Position-Specific Iterative-Basic Local Alignment Search Tool (PSI-BLAST) is usually the first method of choice for the functional annotation of proteins. We carried out the PSI-BLAST analysis on the non-redundant positive dataset of fungal adhesins in a manner like leave-one-out cross-validation (LOO CV), with the cut-off E-value (-e option of blastpgp) of 0.001 and the number of iterations as 3. Each sequence was used as the query sequence once with the rest forming the target database, thus iterating, for each sequence. Herein, no significant hits were obtained for 25 out of 75 sequences, which signifies that homology-based searches alone are not sufficient to identify these proteins.

### Performance of standalone SVM models

**Figure 1:**
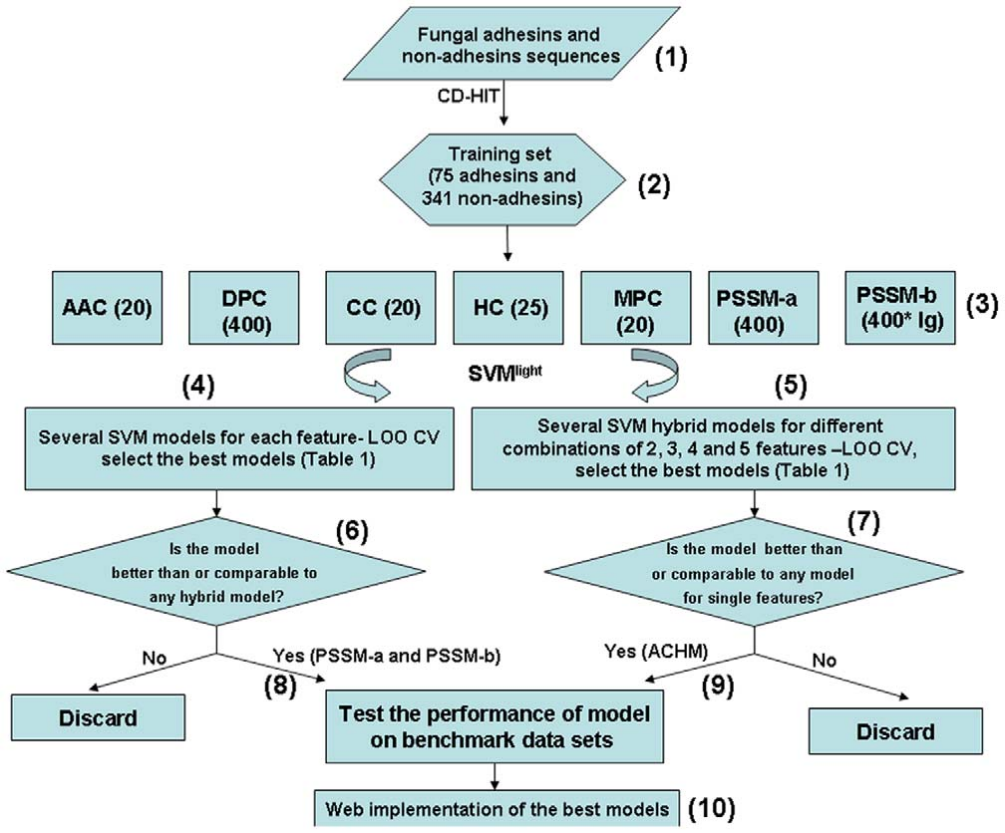
Flowchart of the experimental procedures. (1) A pool of fungal adhesins and non-adhesins sequences was generated from sequence and bibliographic databases (GenBank, UniProt, PubMed). (2) Using CD-HIT, the redundancy of the sequences from both the sets was scaled to 50% threshold, yielding 75 adhesins (positive set) and 341 non-adhesins (negative set). (3) Seven different features of different dimensions (mentioned inside brackets) were extracted using PERL scripts for both the sets. For PSSM-b, lg means lag, i.e. distance along the sequence, for details c.f. [35] (4) LOO CV was done on each of the features and several SVM models with different C and γ generated. The models giving good accuracies and almost equal sensitivity and specificity were selected. (5) Several different combinations of 2, 3, 4 and 5 features were made and LOO CV run on these. Here also the best ones were selected. (6) If the performance of the seven best models trained on different individual features was comparable to or better than the best hybrid models, it was selected for further evaluation. Here the models PSSM-a and PSSM-b were selected. (7) If the hybrid model provided an edge over its constituent individual features or the other hybrid models (in terms of accuracy), it was selected (ACHM) for further evaluation. ACM was another best model amongst the hybrids but offered lower accuracy than ACHM, so was not considered further. (8) & (9) The best SVM models were tested on benchmark data sets. (10) The PSSM-a and ACHM models were implemented on the web server.

We performed LOO CV of Amino Acid Composition (AAC), Dipeptide Composition (DPC), Charge Composition (CC), Hydrophobicity Composition (HC), Multiplet Composition (MPC) and Position-Specific Scoring Matrix (PSSM) based classifiers, trained using the Radial basis function (RBF) kernel (Figure 1). Thereafter, hybrid models using combination of two or more features were also developed. Table 1 depicts the performance of the best SVM classifiers for each module as observed in the LOO CV.

### Composition based SVM classifiers

We obtained an accuracy of 83.65% with both the AAC and DPC-based and 76.68% with MPC-based model. The CC and HC-based models as standalones performed even worse with the maximal accuracy of 73.55% and 73.31% respectively. It is clear that the charge, hydrophobicity and multiplet compositions alone lack sufficient information to provide a good discrimination between adhesins and non-adhesins.

### PSSM profile based SVM classifiers

Apart from encapsulating residue composition, the PSSM profiles capture useful information about conservation of residues at crucial positions within the protein sequence, because in evolution the amino acid residues with similar physico-chemical properties tend to be highly conserved due to selective pressure. PSSM profiles have been employed for training SVMs for a legion of classification problems, like prediction of cyclins [21], nucleic acid binding residues [22], protein subcellular localization [23] etc.

For the model generated with PSSM profiles normalized using the logistic function (PSSM-a), we got a maximum accuracy of 86.29%. For the other model based on PSSM (PSSM-b), initially different lg values ranging from 5 to 40 were tried for Auto-Cross Covariance (ACC) transformation of PSSM profiles but the optimal lg (on the basis of highest accuracy) was obtained as 15. This model gave an accuracy of 81.97%, but the sensitivity for this one (82.66%) was higher than the one generated using logistic function (80%), while the reverse was true about specificities which stood at 87.68 and 81.81% respectively for the PSSM-a and PSSM-b models.

### Performance of hybrid SVM models

With an aim to further enhance the prediction accuracy, we developed and evaluated several hybrid models using different combinations of features. Here we discuss only the best ones obtained in the study, i.e. wherein the hybrid gave some edge over any of the individual features used independently.

#### ACM based classifier

The 60-dimensional input vector for this model comprised of AAC, CC and MPC features. The sensitivity for this classifier stood at 80.00% which was higher than any of the individual features used though the specificity (78.29%) and overall accuracy (78.60%) were lower than the AAC based model.

#### ACHM based classifier

The 85-dimensional input vector for generating this classifier consisted of concatenated features of AAC, CC, HC and MPC. Along with PSSM models, this was one of the best classifiers obtained in the study with an accuracy of 86.05%, sensitivity of 82.66 and specificity 86.80%. Thus this one was indeed an improvement over any of the individual features alone.

Several other hybrids were generated with the PSSM-based classifiers; however these performed only as well as the PSSM-based classifiers, without offering any extra accuracy.

### The best three models: the FaaPred ‘misses’ and ‘hits’

**Figure 2:**
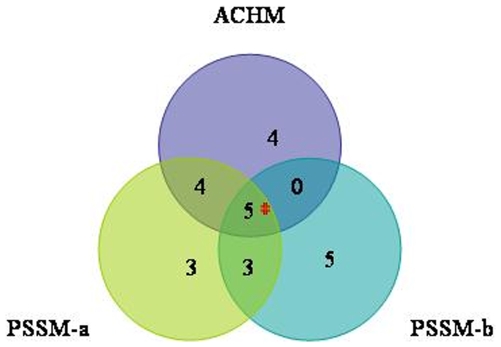
Venn diagram showing ‘misses’ and ‘worst misses’ of the best three SVM classifiers. A good overlap is seen amongst the 24 positives missed out by any one of the best three classifiers (‘misses’), 5 positive sequences (marked with red asterisk) are the ‘worst misses’ which are not predicted by either of the three.

Based on the performance metrics of the various models (Table 1) trained on individual features and combination of features, we selected three best models and evaluated them further. The best models are PSSM-a, PSSM-b and ACHM hybrid model. Intriguingly, we observed a good overlap amongst the positives missed out by these three models in LOO CV (Figure 2). Out of a total of 75 adhesins, there were there were 24 positives (‘misses’) which were missed by at least one of the models and only 5 positives (‘worst misses’) which were missed by all the three classifiers. The rest 51 (‘hits’) were predicted by all the three models. It was imperative to analyze ‘misses’ and ‘hits’ in order to understand if there are any particular features that might explain the occurrence of ‘misses’. Amongst the ‘misses’, there was no specific bias towards any particular species. Further, we analyzed three different aspects of ‘misses’ and ‘hits’- 1) Low Complexity Regions (LCRs) using SEG program [24] (using default trigger window length of 12 and trigger complexity cut-off 2.2) and tandem repeat (TR) regions (with more than 4 amino acid residues, see Methods), 2) AACs after removing the LCRs and TRs and 3) the presence of GPI-anchor using GPI-SOM program [25].

**Table 1:** Performance of different SVM classifiers in LOO CV.

Model	C	γ	Th	SN	SP	Accuracy	MCC
AAC	19	0.001	−0.9	80.00	84.45	83.65	0.557
DPC	2	0.01	−0.7	80.00	84.45	83.65	0.557
CC	0	4	−1.0	64.00	75.65	73.55	0.328
HC	0	0.1	−1.0	68.00	74.48	73.31	0.346
MPC	2	0.001	−1.0	77.33	76.53	76.68	0.439
ACM	0	0.00001	−1.0	80.00	78.29	78.60	0.479
ACHM	20	0.001	−0.8	82.66	86.80	86.05	0.610
PSSM-a	22	4	−0.6	80.00	87.68	86.29	0.604
PSSM-b	60	0.0001	−0.6	82.66	81.81	81.97	0.541

Th–Threshold, SN–sensitivity, SP–specificity, MCC–Matthews Correlation Coefficient.

LCRs and TRs were more abundant in ‘hits’ and had relatively higher content of Ser, Thr and Val (Figure S1). In ‘misses’, the LCRs and TRs were less prevalent and had relatively higher contents of Gln, Asn, Pro, Gly and the charged amino acids Asp, Lys, Arg. 39 ‘hits’ and only 8 ‘misses’ showed the presence of TRs while 51 ‘hits’ and 16 ‘misses’ showed the presence of LCRs.

After removing the LCRs and TRs, we analyzed average AACs within the ‘misses’ and ‘hits’ sequences (Figure S2). The content of Thr was still remarkably higher in ‘hits’ than in ‘misses’. The contents of Ala, Gly, Leu and Asn and charged amino acids Asp, Glu, Arg and Lys were higher in ‘misses’ than in ‘hits’.

In the analysis for the presence of C-terminal GPI-anchors, it was observed that 35 hits, only 2 misses and none of the ‘worst misses’ showed the presence of the anchor. The enrichment of the sequences with GPI anchors within ‘hits’ indicates that the ‘misses’ may adopt different attachment signals than those of hits.

### Receiver Operating Characteristic (ROC) plot

**Figure 3:**
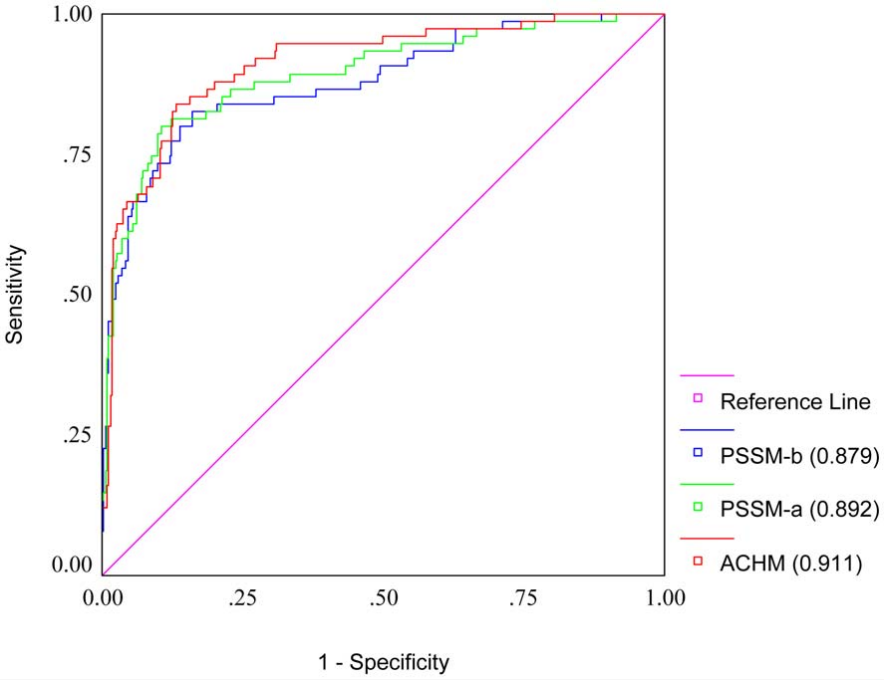
ROC curves of the different SVM classifiers. ROC plot of SVMs based on different protein sequence features depicting relative trade-offs between true positive and false positives. The corresponding Area Under Curve (AUC) is given in brackets in the legends.

The ROC curve (Figure 3) was used to evaluate the threshold-independent performance of the three best models and shows the trade-off between true positive rate (sensitivity) and false positive rate (specificity) over their entire range of possible values. The ACHM classifier had Area Under Curve (AUC) of 0.911 while PSSM-a and PSSM-b models of 0.892 and 0.879 respectively. Thus the three best classifiers chosen performed far better than the AUC threshold of random prediction, i.e. 0.5. This confirmed the effective discriminative power and robustness of the models.

### Performance on benchmarking datasets

Table 2 lists the performance of the three classifiers on the independent positive and negative test datasets. This was assessed at the default thresholds obtained by cross-validation studies (see Table 1), however for practical purposes, the higher the scores, the higher is the confidence level of prediction. The remarkably fair accuracies of the three classifiers for both the datasets demonstrate its efficiency and justify its use for practical application.

**Table 2:** Performance on benchmark datasets.

Model	Positive set (32)	Negative set (310)
ACHM	31	263
PSSM-a	32	280
PSSM-b	32	264

The numbers show the correctly predicted sequences out of the total shown in the first row, 32 for the positive set and 310 for the negative set.

### Web Implementation

**Figure 4:**
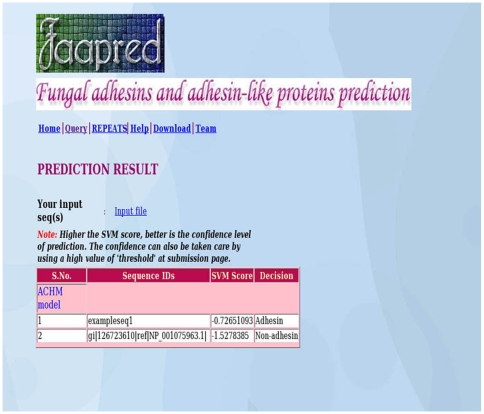
Snapshot of FaaPred web server sample output. The web server predicts fungal adhesins based on the two best classifiers, namely based on PSSM profile (PSSM-a) and the hybrid classifier: ACHM. The two classifiers may be chosen together for a comparative prediction. The server accepts FASTA formatted sequences and allows user defined thresholds of prediction, ranging from −1.5 to 1.5.

The prediction algorithm presented in this study is implemented as a freely accessible web server at http://bioinfo.icgeb.res.in/faap (Figure 4). The web server is hosted on a T1000 SUN server using Apache. PHP is used for server side scripting. The background running programs for calculation of compositional properties and PSSM profiles and their conversion to SVM format are done using PERL scripts. The program predicts adhesins using the ACHM and PSSM-a classifiers. Since the PSSM-b classifier uses 6000 variables, the predictions are extremely time-intensive while the performance is as good as the other two classifiers; this model has not been put up on the web-server. The input sequences are provided in the FASTA format and the program allows the user to perform prediction at thresholds ranging from -1.0 to 1.5. The output returns the sequence ID, the SVM score and the decision of the model regarding the sequence based on the threshold chosen.

### Sensitivity of the SVM models for species not represented in training datasets

In order to assess if the FaaPred approach could be applied to species which are not represented in the training dataset (i.e. to check if the method could be used to predict novel fungal adhesins), we excluded adhesin and non-adhesin *Candida albicans* sequences and the closely related *Candida dubiliniensis* from the training datasets and then generated three new SVM models for ACHM, PSSM-a and PSSM-b classifiers. Thereafter we checked the performance of these models on the excluded adhesins. The ACHM model was able to predict 12 out of 14 adhesins while both the PSSM-a and PSSM-b could correctly predict 11 and 14 adhesins respectively. The promising results obtained from this analysis demonstrate that the FaaPred may be applied to species not included in the training sets.

### Application of FaaPred for whole proteomes

We used the ACHM and PSSM-a models to scan *Schizosaccharomyces pombe* proteome (http://www.sanger.ac.uk/Projects/S_pombe). We used a stringent SVM score threshold of 0.5 to reduce the number of false positives. The ACHM and PSSM-a models reported 33 and 35 positives respectively. The complete list of predicted adhesins includes 10 ‘sequence orphans’ or ‘dubious’ proteins and few proteins annotated as ‘conserved fungal proteins’. Of special interest were 16 positives common to both the models (Table S1). Two amongst these are Mam3 and Map4 proteins which are known for their role in adhesion. Ten of these are annotated as cell wall glycoproteins (one as cell wall organisation protein). We term these as ‘adhesin-like’ proteins which are the candidate adhesin proteins that could be investigated for their role in adhesion. Though the exact role in adhesion is not established for these, interestingly another study [26] based on comparative phylogenetics had previously suggested six of these ten sequences as potential adhesins (c.f. Table S1). Some false positives also appear in the list. An optimum experimental strategy would include considering the total number of proteins to be characterized, prioritizing proteins with other complementary evidence (such as subcellular localization or expression data) while keeping the number of false positives as low as possible. We also performed the above analysis after excluding *S. pombe* sequences from the training sets and generating new models and obtained almost similar results. In this case, we obtained 33 positives for both the models of which 15 are common to both and are the same ones as discussed above.

## Discussion

We developed several SVM-based models using compositional properties as well as PSSM profiles to facilitate the identification of fungal adhesins. The ACHM model emerged as the best classifier followed by the two PSSM models and also performs reasonably faster than the latter which require the generation of PSSM profiles for the input sequences. However one PSSM model, i.e. PSSM-a has been provided on the web server to serve as a complementation to the ACHM model.

The analysis of the prediction ‘misses’ and ‘hits’ for the SVM models developed in the study reflects distinct AACs within the entire protein and low-complexity regions. More GPI-anchors are predicted in the ‘hits’ as compared to the ‘misses’. One of the ‘misses’, namely WI-1/Bad1 adhesin, is known to utilize an alternative mechanism for cell wall attachment. The protein is secreted into the external medium and subsequently attached to the cell wall exterior by non-covalent binding to chitin chains- a process that requires its tandem repeat domains [27]. There are other alternative ways too for cell wall attachment in fungi. Proteins with internal repeats (Pir proteins, which are a group of non-adhesive proteins) become covalently attached to the cell wall sugar molecules directly through glutamine residues within their tandem repeat domains [28]. Some proteins are non-covalently associated with the cell wall polysaccharides or ionically bound to the multiple negatively charged groups like phosphodiester groups in the O- and N-linked carbohydrate side chains of cell wall glycoproteins [29]. Though these mechanisms are not yet established for adhesins, it is plausible that the ‘misses’ could be having any of these or some other alternative mechanisms of cell wall attachment, making them distinct from the ‘hits’. This also highlights that the SVM classifiers developed in the study overcome the limitation of the presence of GPI anchor to a great extent as there are only five misses (‘worst misses’) which are not predicted by either of the three classifiers.

The overall adhesion determinants resulting from the differential overall compositions as well as within the LCRs and thereby the charge and hydrophobicity characteristics of the ‘misses’ might be quite different from those of ‘hits’, accounting for the inability of one or more classifiers to predict them. The enrichment of both positively and negatively charged amino acids within the ‘misses’ is intriguing and it is tempting to speculate that this could provide basis for a unique way of cell wall attachment, which may be investigated further.

The adhesin dataset used in the study represents adhesins from 29 different species with diverse taxonomic positions (Figure S3), however this certainly does not represent adhesins from all the fungi. This restriction stems from the fact that as opposed to bacteria, there is a paucity of available fungal adhesin sequences. The reason for the successful performance of the models on sequences of species not included in training, as seen for both *Candida* species and *S. pombe* is that SVMs gather sufficient information to create classification model based on only a small set of the training examples. Though we have tested the sensitivity of the approach on species not represented in training sequences, the true sensitivity towards extremely divergent species may only be tested when such sequences are available in future. The prediction method developed in the study can expedite the discovery of adhesins in fungi and needs to be judiciously used, keeping the SVM scores as well as other complementary evidence into consideration. Thus FaaPred has the potential to be used for scanning of adhesin-like properties in fungal proteomes. In future, availability of additional adhesin sequences with a better representation of different fungal species and inclusion of more properties would further enhance the accuracy of the program.

## Methods

Figure 1 provides an overview of our experimental strategy and is described below in detail.

### Generation of datasets for SVM training

Different keywords like ‘adhesin’, ‘flocculin’, ‘agglutinin’, with the limiting filter of taxonomy as fungi were used to compile a raw pool of fungal adhesin sequences from sequence (Genbank and UniProt) and bibliographic databases (PubMed). Proteins with known intracellular locations, such as nucleus, cytoplasm, mitochondria, endoplasmic reticulum etc. were collected and assigned to the non-adhesin set. Both the sets were filtered for hypothetical proteins and protein fragments.

The redundancy in both the sets was scaled down to 50% using the CD-HIT program [30]. Hereupon, we had two sets containing full-length and well-annotated sequences of 75 adhesins and 341 non-adhesins from fungi (Datasets S1 and S2 respectively).

### Benchmark dataset for testing

In order to examine the unbiased prediction efficiency of our best SVM models, we tested their performance on independent datasets not used in training or testing cycles. While one test dataset consisted of 32 fungal adhesins, the other had 310 non-adhesins from different fungi species (Datasets S3 and S4 respectively).

### SVMs and SVM^light^

First pioneered by Vapnik in 1995, SVM is a supervised machine learning method which delivers state-of-the-art performance in recognition and discrimination of cryptic patterns in complex datasets [31]. SVM is used in conjunction with kernel functions which implicitly map input data to high dimensional non-linear feature space. SVM then constructs a hyperplane separating the positive examples from the negative ones in the new space representation. To avoid over fitting, SVM chooses the Optimal Separating Hyperplane (OSH) that maximizes the margin i.e. the minimal distance between the hyperplane and the training examples [32]. The selected data points supporting the hyperplane are called support vectors.

We implemented SVM using SVM^light^ package (http://svmlight.joachims.org) which allows us to choose a number of parameters and kernels (e.g. linear, polynomial, radial basis function, sigmoid or any user-defined kernel). In this study we used the RBF kernel. For detailed descriptions of SVM please refer [33].

The positive class for building SVM models in this work implies adhesins (from fungi) while the negative class signifies non-adhesins (from fungi). We performed training testing cycles using in-house shell and PERL scripts. We used RBF kernel to train and test our SVM models. The values of λ and regularization parameter C were optimized on the training datasets by cross-validation. The overall strategy was to choose the best parameters in a way so as to maximize accuracy along with nearly equal sensitivity and specificity, wherever possible.

### Leave-one-out cross validation

This is deemed as the most objective and rigorous mode of evaluation wherein one dataset sequence is singled out for testing, while the rest are used to generate the model. This iterates on each sequence till each sequence becomes the testing data exactly once. This is a stringent case of n-fold cross-validation where n equals the total number of sequences. The best parameters (λ and C) as measured by the various performance measures (explained below) are taken and then averaged to get overall assessment of the model.

### Classifier performance metrics

To evaluate the accuracy of SVM classifiers developed in cross-validation cycles, we used the following four measures:

1. Sensitivity: percentage of adhesin protein sequences that are correctly predicted as adhesins.
2. Specificity: percentage of non-adhesin protein sequences that are correctly predicted as non-adhesins.
3. Accuracy: percentage of correct predictions out of total number of predictions.
4. Matthews correlation coefficient (MCC): a measure of both sensitivity and specificity, MCC = 0 indicates completely random prediction, while MCC = 1 indicates perfect prediction.

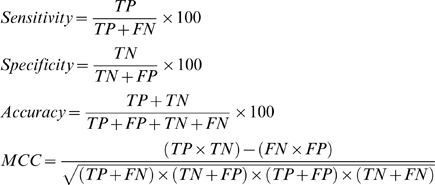



### Feature extraction

*Amino acid composition (AAC)*: It is the fraction of each of the 20 amino acids present in a protein sequence. This generates an input vector of 20 dimensions.

*Dipeptide composition (DPC)*: It is the fraction of a dipeptide divided by the total number of possible dipeptides. This yields a training vector of 400 dimensions.

*Charge composition (CC)*: It is the fraction of charged amino acids divided by the length of the protein. Moments (Mr) of the positions of charged amino acids (R, K, D and E) from order 2–19 are calculated using the expression:
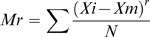



where Xm is the mean of all positions of charged amino acids, Xm = 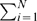
Xi/N; Xi is the position of i-th charged amino acid while N is the total number of charged amino acids in the sequence. The fractions of positively and negatively charged amino acids together with 18 moments yield a fixed length input vector of 20 dimensions.

*Hydrophobicity composition (HC)*: The amino acids may be classified into five groups on the basis of their hydrophobicity properties [34]. Moments of the positions of the five groups were calculated using the same formula as above with r varying from 2 to 5. The fractions of five groups together with 20 moments provide a fixed length input vector of 25 dimensions.

*Multiplet composition (MPC)*: Multiplets are homopolymers (X)n, where X is any amino acid repeated n times with n≥2. This generates a 20-dimensional input vector.

*Position specific scoring matrix (PSSM) profile*: This was obtained by performing PSI-BLAST against SwissProt database (release 57.3) at the default E-value (-h option of blastpgp) of 0.001 with three iterations. The matrix contains 20×N elements, N being the length of the query sequence, and each element represents the frequency of a particular residue substitution at a specific position in the alignment. To generate input vectors of fixed length for SVM training, this was transformed in two ways. First, where the PSSM matrix was normalized between 0 and 1 using the following logistic function: 
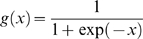



Where x is the raw value in PSSM profile and g(x) is the normalized value of x. Following this, the normalized matrix is organized into a composition matrix of fixed length pattern of 400 (20×20, for each amino acid, there are 20 substitution scores from normalized matrix).

Second, where the PSSM matrix was subjected to auto-cross covariance (ACC) transformation (for details consult Dong *et al*. [35]) at different lags lg varying from 5 to 40. This generates input vectors of fixed dimensions, i.e. 400*lg, where 20*lg are auto-covariance (AC) variables while 380*lg are CC (cross-covariance) variables.

### ROC plot

Statistical Package for Social Sciences (SPSS) software for Windows version 11.5.0 was used to obtain the ROC plot [36] for the SVM classifiers developed in the study.

### Tandem amino acid repeats analysis

Tandem amino acid repeats are stretches of a single amino acid repeated consecutively. All such repeats longer than four amino acids were discovered in the ‘misses’ and ‘hits’, with a PERL script using the regular expression “[ACDEFGHIKLMNPQRSTVWY]{4,}”.

## Supporting Information

Figure S1 The composition of low-complexity regions (LCRs) in ‘hits’ and misses'. This is a tiff file.
(0.05 MB TIF)

Figure S2 Amino acid compositions of ‘hits’ and ‘misses’ after removing LCRs and TRs. This is a tiff file.
(0.06 MB TIF)

Figure S3 The taxonomic positions of the fungal species included in training sets. This is a tiff file.
(0.20 MB TIF)

Table S1 The 16 common positives predicted as adhesins and adhesin-like proteins during whole genome scan of S. pombe. This is a ms excel file.
(0.01 MB XLS)

Dataset S1 Positive dataset. This consists of 75 sequences of fungal adhesins used for training the SVMs. This can be viewed using any text editor like wordpad.
(0.07 MB TXT)

Dataset S2 Negative dataset. This consists of 341 sequences of fungal non-adhesins used for training the SVMs. This can be viewed using any text editor like wordpad.
(0.22 MB TXT)

Dataset S3 Blind test dataset for positives. This consists of 32 fungal adhesin sequences not used in training or testing. This can be viewed using any text editor like wordpad.
(0.04 MB TXT)

Dataset S4 Blind test dataset for negatives. This consists of 310 non-adhesin sequences from fungi. This can be viewed using any text editor like wordpad.
(0.17 MB TXT)
