# Supplementary figures and images for: FaaPred: A SVM-Based Prediction Method for Fungal Adhesins and Adhesin-Like Proteins
Source: PLoS One. 2010 Mar 15;5(3):e9695. doi: 10.1371/journal.pone.0009695 (PMC2837750; doi:10.1371/journal.pone.0009695)

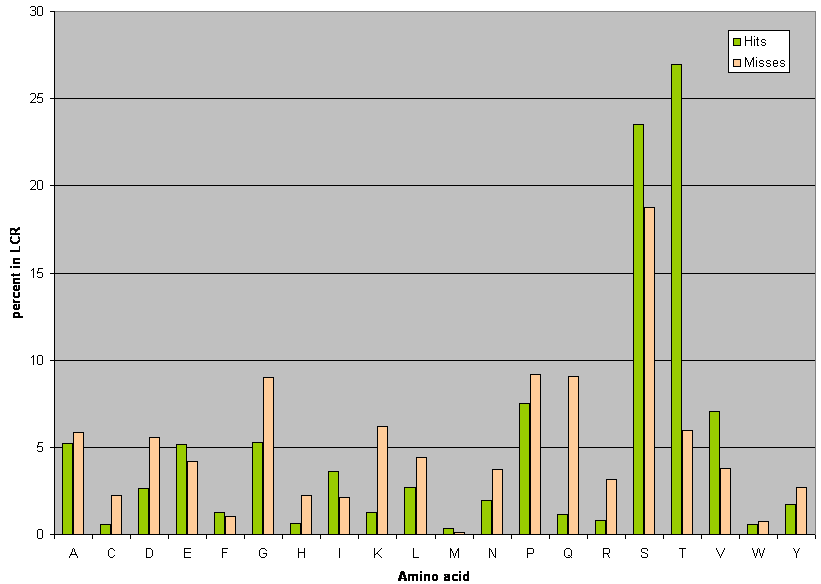

Supplement: Figure S1 — The composition of low-complexity regions (LCRs) in ‘hits’ and misses'. This is a tiff file. (0.05 MB TIF) [file pone.0009695.s001.tif]

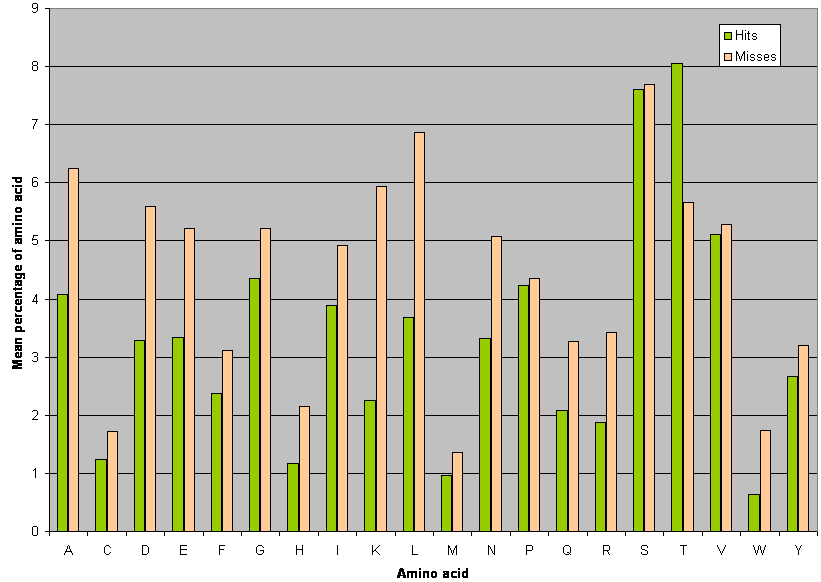

Supplement: Figure S2 — Amino acid compositions of ‘hits’ and ‘misses’ after removing LCRs and TRs. This is a tiff file. (0.06 MB TIF) [file pone.0009695.s002.tif]

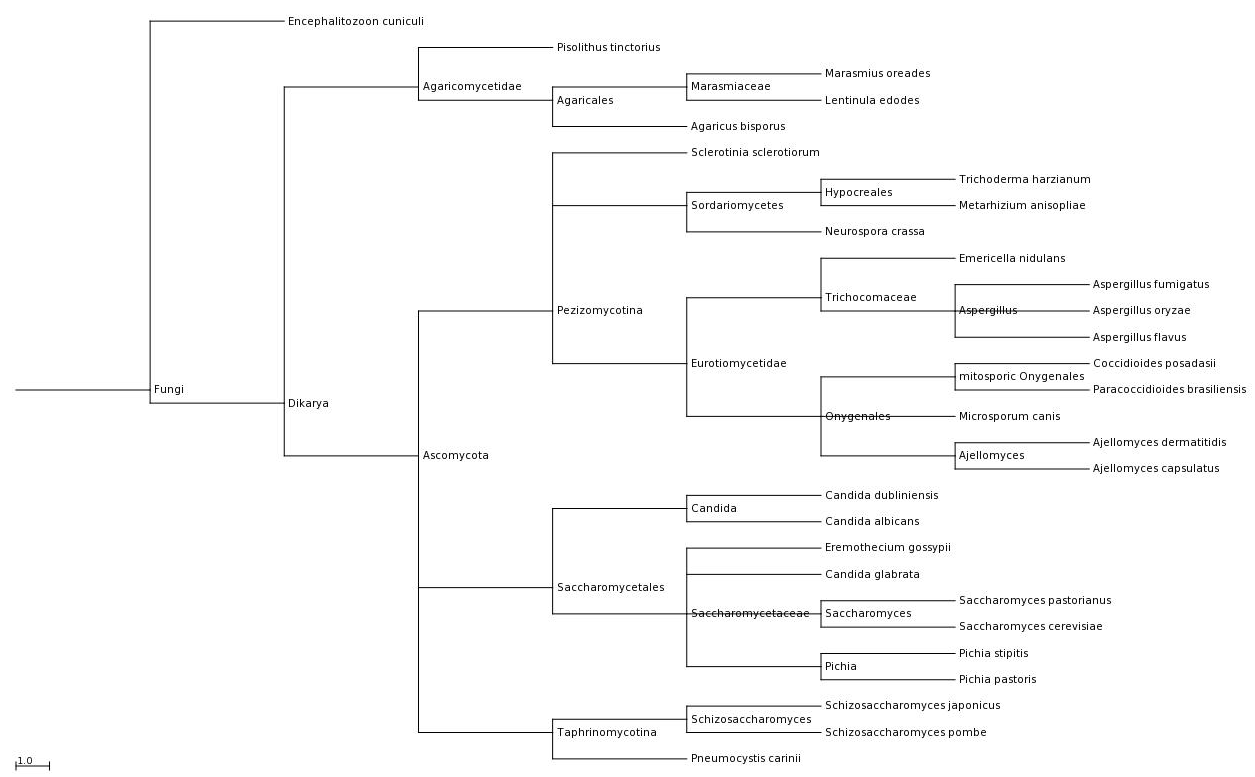

Supplement: Figure S3 — The taxonomic positions of the fungal species included in training sets. This is a tiff file. (0.20 MB TIF) [file pone.0009695.s003.tif]
